# Perspectives of Three African American Chemists: Reflections on Careers, Experiences, and the Future

**DOI:** 10.1021/acs.analchem.2c01905

**Published:** 2022-07-19

**Authors:** Isiah M. Warner, Willie E. May, James W. Mitchell

**Affiliations:** † Department of Chemistry, Louisiana State University, Baton Rouge, Louisiana 70803, United States,; ‡ Division of Research and Economic Development, Morgan State University, Baltimore, Maryland 21251, United States; § Department of Chemical Engineering, Howard University, Washington District of Columbia 20059, United States

## Abstract

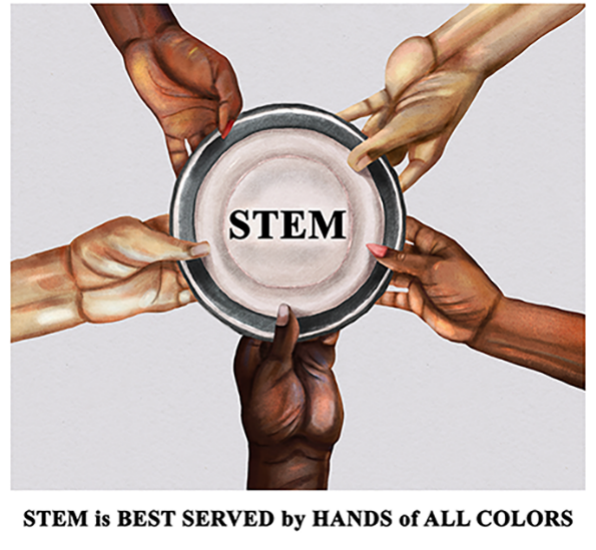

Three African American analytical chemists, whose primary research careers have focused in the respective sectors of academia, government, and industry, have come together to provide personal perspectives on parameters that have impacted their careers as well as to provide their perceptions of the current and future status of African Americans in the overall science, technology, engineering, and mathematics (STEM) enterprise, and the more specific field of analytical chemistry. The authors, having ∼150 years of combined experiences, reflect on the past status and contemplate future advances for African Americans in STEM. The most important factors during their formative years that underpinned their success over the long-term are enumerated. Particularly cited are the distinct features within the Historically Black Colleges and Universities (HBCU) environment that placed them solidly on a path toward successful careers. The Grand Challenge now and for the foreseeable future, reversal of the dysfunctional metropolitan public-school systems, is cited and the only perceived light in the tunnel for addressing this issue is mentioned. Finally, recommendations are made for the future where diversity within the STEM enterprise will be a prerequisite for U.S. competitiveness in our global society

## Introduction

To provide an overall perspective of the experiences of African American scientists in academia, industry, and government, three senior African American analytical chemists have come together to provide unique perspectives on their careers. All three are well-respected leaders in academia, government, and industry.

In addition to celebration of each of these three African American chemists for their scientific accomplishments, they are also celebrated as trailblazers for promoting underrepresented groups in chemistry and as mentors to minorities and women in science, technology, engineering, and mathematics (STEM).

While each spent most of their careers in different sectors, all three are currently working in academia. In addition, all three have attributed their successful careers to foundations laid by their parents, elementary and high school teachers, as well as their college professors. They further expound on these individual factors in this perspective.

Although these chemists have been aware of each other over the years, their careers have developed independently over the last half century. Interestingly, they show many parallels in time and experience. All have acknowledged that they have faced racism during their formative years of growth and careers but have decided not to focus on that aspect in this perspective. Inevitably they have converged at several points, such as this moment, when they have come together to offer a unique perspective on their past and the future of STEM in America with a focus on African Americans in the field of analytical chemistry.

## Who Are They?

While most articles provide biographies at the end of manuscripts, these authors elect to provide abbreviated biographies near the beginning of this manuscript to specify a context on which many statements are based and conclusions drawn. In addition, this approach allows the authors to a priori provide the reader with aspects of their backgrounds that they believe contribute to their individual successes and thus to the conclusions drawn in this manuscript.

## Dr. Isiah M. Warner

Dr. Isiah Manuel Warner is a recently retired Boyd Professor of the Louisiana State University System (highest possible rank), Philip W. West Professor of Chemistry, and Howard Hughes Medical Institute Professor. He has won numerous awards for his research as well as for his contributions to education and mentoring.
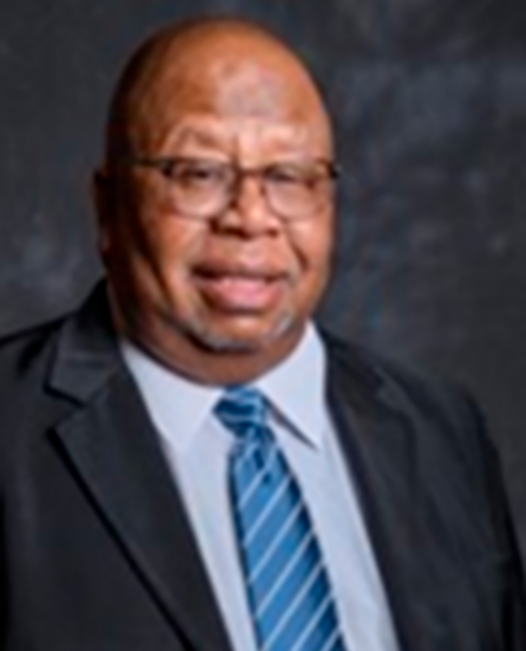


Dr. Warner was raised in the small town of Bunkie, Louisiana (population ∼5000). He has always had an innate curiosity about the world around him. In fact, he states that he performed his first “analytical chemistry experiment” at the age of two when he orally sampled kerosene to determine how this odd smelling liquid could produce light. This landed him a stay in the hospital and an early recognition that oral sampling of chemicals is not an acceptable analytical technique. To satisfy his curiosity and stave off his oral sampling method, his parents kept him away from chemicals for a while but later purchased his first chemistry set at the age of 12.

In his youth, Dr. Warner worked in the cotton fields and hated it. For that reason, there were some in his community who viewed him as indolent. However, his grandmother never did. Dr. Warner recounts one aspect of his grandmother’s support:

“One day, I came home complaining about the hot and humid Louisiana weather after working in the cotton fields for 10 h. The temperature was often in the mid 90 °F and the percent humidity also in the mid-90s. While weary and tired, I told my grandmother that I would not do that kind of work the rest of my life. She then asked, “What are you going to do, be a teacher?” I said, “That sounds good to me!” Thus, his grandmother was the first person to suggest that he might teach and mentor students someday. He has worked hard to fulfill that dream placed early in his mind by his maternal grandmother. As an assiduous and successful chemist and teacher, no one can validly call him indolent today.

Dr. Warner is the eldest of three siblings and was the first in his immediate family to obtain a high school diploma. He graduated valedictorian of his 1964 Carver High School class in Bunkie, Louisiana. After high school, he participated in a summer institute at Southern University (a Historically Black Colleges and Universities (HBCU)), which based on his performance allowed him to skip the first year of college chemistry. He attended Southern University in Baton Rouge, Louisiana, on a full scholarship earning a B.S. in chemistry and graduating Magna Cum Laude. After college, he worked 5 years for Battelle Northwest (Prime contractor for the Atomic Energy Commission). However, he recognized that in that arena, a B.S. degree would not advance his career to the level he desired. Therefore, he left Battelle to pursue a Ph.D. in Chemistry from the University of Washington, Seattle, graduating with a Ph.D. in 3 and a ^1^/_2_ years and a near perfect GPA, from a program that averaged more than 5 years for a Ph.D.

Dr. Warner’s career in the academic arena spans close to 45 years, having served at Texas A&M University (5 years), Emory University (10 years), and Louisiana State University (∼30 years). He has held an endowed professorship at both Emory and Louisiana State University and was awarded early tenure and promotion at Texas A & M University.

His research as an analytical/materials chemist has focused in the areas of fluorescence spectroscopy, organized media, separations science, and ionic liquid chemistry, with a focus on applications to solid phase ionic materials for the latter. He has more than 385 refereed publications and has contributed more than 500 invited lectures all over the world in the areas of analytical and materials chemistry and education.

Professor Warner has combined his work endeavors into the areas of research, mentoring, and education. The latter has aided him in mentoring hundreds of students, postdoctoral associates, and faculty to successful careers in STEM fields. He is well-known in the STEM field for his research and is also among the best-known mentors in the world, the latter of which is an area for which he has won as many awards as for his science. He takes great pride in both accomplishments.

Dr. Warner’s educational philosophy maintains that “A student’s background is determined by their past. However, hard work and aptitude is what determines their future.” He believes that his mentorship has been a prime component in his helping students to reach their maximum educational potential.

Professor Warner has produced 68 Ph.D.s and 8 Masters’ degree students. He takes great pride in the fact that more than half of his Ph.D. students have been women, and more than a third under-represented minorities. Similar numbers are also evident for his postdoctoral associates and visiting faculty.

In addition to his many research awards, he has also won numerous regional, national, and international awards for education and mentoring. As examples of awards for research, education, and mentoring, he is the recipient of (1) a Presidential (President Reagan) Young Investigator Award, (2) ACS Award for Analytical Chemistry, (3) ACS Division of Analytical Chemistry Award in Education, (4) Presidential (President Clinton) Award for Excellence in STEM Mentoring, (5) Fellow of the American Academy of Arts and Sciences, (6) Fellow of the National Academy of Inventors, (7) SEC Professor of the Year, (8) Fellow of the American Chemical Society (inaugural class), (9) Fellow of AAAS, and (10) International Journal Nature Award for Mentoring.

Professor Warner is currently working on a mentoring book that describes his mentors as well as the persons he has mentored. As a mentor, he advises future STEM scientists to “Be confident in your own abilities. Do not let negativity from any source turn you away from science if science is your true love.”

## Dr. Willie E. May

Dr. Willie Eugene May is the oldest of three children born to Willie Edward May, a WWII veteran and entrepreneur and Rubie Daniels May, an early childhood educator.
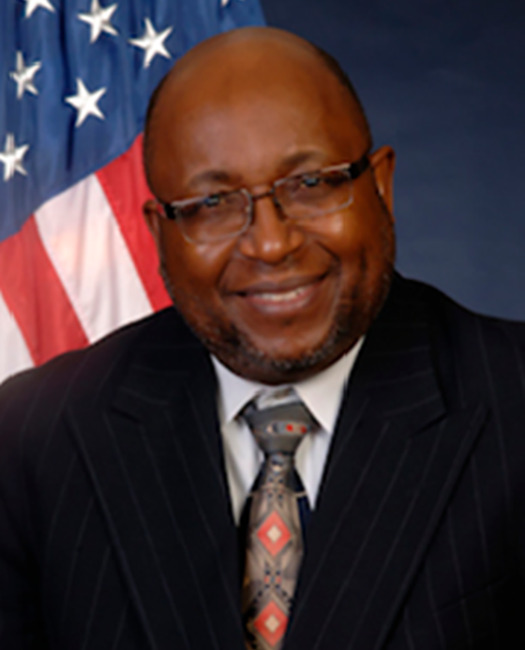


Both his parents instilled a great work ethic and ambition in him. He was a naturally gifted scholar and loved learning but loved athletics more. In fact, his father thought baseball would be Dr. May’s path to success. However, his mother insisted “my boy is going to college.”

Dr. May’s high school chemistry instructor, Mr. Frank Cook, inspired his interest in chemistry. He took him and a few others under his wing and exposed them to a college-level chemistry curriculum in grades 10–12.

With a strong aptitude for college work and stellar grades, Dr. May applied for college with hopes of attending Howard University. Due to a mistake in his high school registrars’ office, his application was lost and only located after Howard’s admissions deadline had passed. His high school principal (R. C. Johnson, General Colin Powell’s father-in-law) made some calls and arranged a full scholarship for him to attend Knoxville College, a small HBCU in East Tennessee. He excelled there and 4 years later, graduated Summa Cum Laude, with a B.S. in chemistry.

Upon graduation, Dr. May had full fellowship offers to attend Harvard, Illinois, and the University of Tennessee. He entered the workforce instead and became the first person of color to be employed in the Analytical Chemistry Division at the Oak Ridge Gaseous Diffusion Plant in Oak Ridge, Tennessee. During his 3-year tenure, while he was not the subject of overt racism, the micro aggressions and lack of advancement opportunities caused Dr. May to seek employment elsewhere.

With the support of his Knoxville College mentor, Dr. Jesse James, he relocated to the Washington, DC area and found a much better match at the National Bureau of Standards (now NIST). He began a 1-year term appointment there on July 6, 1971. While the racial demographics were very similar at both Oak Ridge and NBS, he found the latter to be more of a “scientific meritocracy”. This was exactly the type of environment he sought. After 1-year, he obtained a permanent appointment and remained there for another 44.5 years.

After his first 3 years at NBS/NIST, he was encouraged to pursue a Ph.D. at the University of Maryland, College Park. He worked full time while carrying a full course load. He completed his course work and his dissertation “The Solubility Behavior of Polycyclic Aromatic Hydrocarbons in Systems” in 4 years. During the period that he was working and attending school, he became a key member of the team charged with determining biogenic levels of petroleum-hydrocarbons in Prince William prior to completion of the Trans-Alaskan Pipeline. Liquid chromatographic methodology developed by Dr. May was crucial to success of this pioneering effort. Data from this study was later used for “Damage Assessment” when the Exxon Valdes ran aground in Prince William Sound in the 1980s.

He rose through the ranks at NBS/NIST and worked and excelled at every research and management level within the organization. Though the first lessons he learned about management came growing up in his neighborhood in Birmingham in the 1950’s and 1960’s, based on his scientific stature, he became the first and only African American to be elected to the 18-member International Committee on Weights and Measures (CIPM) that oversees the world’s measurement system.^24^ He served in this capacity for more than a decade, 8 as its Vice President.

Dr. May was a pioneer on use of high-performance liquid chromatography as a quantitative analytical tool. His research activities were focused in the areas of trace organic analytical chemistry with emphasis on environmental and clinical applications and determination of physicochemical properties of organic compounds. His work is described in more than 90 peer-refereed publications, and he has given more than 300 invited lectures at conferences and symposia around the world.

In 2014, Dr. May was nominated by President Obama and in 2015 unanimously confirmed by the Senate (93–0–7) to become Under Secretary of Commerce for Standards and Technology and NIST Director. His swearing in was the second proudest day of his professional career. The proudest day occurred when he was a first ballot selection to become a member of the NIST Gallery of Distinguished Alumni (“NIST Wall of Fame”) by a panel of his former peers only 9-months after his retirement.

After retiring from NIST, Dr. May was invited back to his alma mater, University of Maryland College Park, to serve as Director of Major Research and Training Initiatives, within the College of Computer, Mathematical, and Natural Sciences. His duties involved developing new relationships and expanding existing partnerships with corporations, foundations, and government agencies. Part of his duties included meeting and greeting potential donors at major sporting events. Although he was successful and enjoyed his position, he felt that he needed to give back to the larger African American community that had nurtured and facilitated his early success, stating:

“In reflection, I realized that I owed so many people who had struggled in the background to provide me with career opportunities that I could never have dreamed of while growing up there in Birmingham during the 1950s and early 1960s. I began to think that the best way to repay some of the debt that I owed was to work at an HBCU and try to share some of my knowledge, experiences, and contacts that I had accumulated over the course of my very improbable career.”

Dr. May accepted a position at Morgan State University in Baltimore, MD, where he currently serves as Vice President for Research and Economic Development and Professor of Chemistry. He is working to aggressively increase the quality and quantity of research outputs, facilitate increased tech transfer and entrepreneurship among both faculty and students, and better connect research across “Maryland’s Preeminent Public Urban Research University” to community needs. He is also leading Morgan’s efforts for ascension to R-1 research status by the end of this decade.

Dr. May is an award-winning scientist, leader, and mentor. He advises future leaders to

- Expect excellence.
- Support a collegial, collaborative, and fair work environment.
- Value a strong work ethic.
- Empower others.
- Be strategic and take risks.
- Always remember where you came from, reach back, and pay forward.

Dr. May advises the next generation of black scientists and engineers to

“Find something that you are/or can be good at and can remain passionate about. When you find that true calling, you will never have a job. You will instead have discovered the path to a rich and rewarding career!”

## Dr. James W. Mitchell

Dr. James W. Mitchell is a materials characterization scientist and chemical processing engineer who contributed to development and research on purification, processing, fabrication, and characterizations of optical waveguides and nanomaterials. As a result of his contributions in these areas of R&D, he is a member of the National Academy of Engineering.
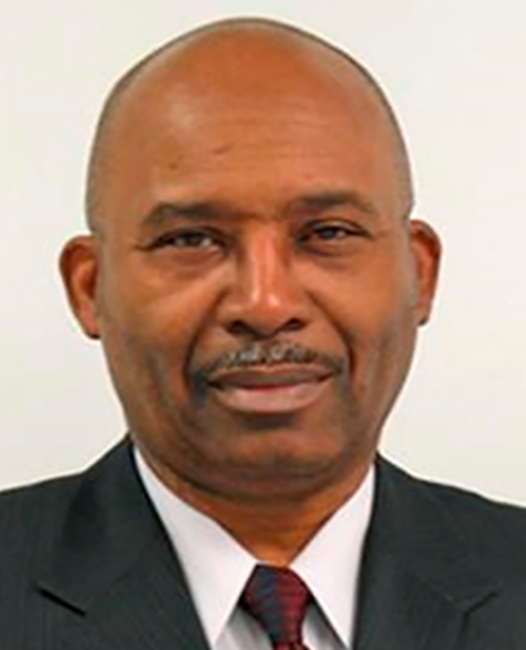


Dr. Mitchell is the first born and only son of the five children of Willie and Eunice Mitchell. As a result of his parents’ instilling confidence, self-respect, a hard work ethic, and discipline, he excelled scholastically. His love for chemistry was inspired in 1960 when he participated in a high school summer program that was funded by the NSF and held at North Carolina Central University, an HBCU in Durham, NC.

Over the summer, a Ph.D. chemist engaged the students in hands-on experiments. The students were guided to electrochemically deposit copper metal films and to grow single crystals of copper sulfate. Dr. Mitchell found those to be exceptionally exciting experiments at the time. When the professor showed Dr. Mitchell how to balance chemical equations mathematically using the oxidation reduction method, that is when he knew he was hooked on chemistry for life.

In addition to his love of science, Dr. Mitchell’s success can be attributed to a strong drive to achieve scholastically in general. He related a time when he fell behind the other students in school:

“In the second grade, the measles caused me to miss school for 2 weeks. When I returned, the other students were reading out loud, but I was not able to do that. I remember studying diligently to catch up and I learned to read well.”

With his love of science encouraged, Dr. Mitchell knew he wanted to attend college and major in chemistry. He decided to attend the Agricultural and Technical State University of North Carolina at Greensboro. Although his family could not afford to pay for his college education, he managed the cost of college with a small tuition scholarship, a National Defense Student Loan, and an on-campus job. Dr. Mitchell graduated from NC A&T State University in 1965.

While pursuing his undergraduate degree, Dr. Mitchell was inspired to continue his education. He completed his Ph.D. in Analytical Chemistry at Iowa State University in 1970.

Dr. Mitchell decided to initiate his career with a U.S. agency or company. He received 14 invitations for employment interviews, resulting in 13 job offers. When he became aware that AT&T Bell Laboratories at Murray Hill, NJ, was widely acknowledged as the most renowned research corporation on planet Earth, he accepted that offer and reported to work on March 31, 1970.

By focusing his research and technology development work on processing, purification, and characterization of optical waveguide raw materials and optical fibers, Mitchell rose quickly through the ranks to become Head of the Analytical Chemistry Research Department in 1975. During the next decade under Mitchell’s leadership, the department obtained “world-class” status in telecommunications materials characterization research and processing technology. His department consisted of 19 Ph.Ds., 5 Post Docs, 13 M.S.s, 5 B.S.s, and 3 TAs.

Dr. Mitchell served in high level administrative positions over the course of his career. His corporate career marked several firsts for African Americans at AT&T Bell Laboratories including the first Bell Laboratories Research Fellow, the first Director of Research, and the first Vice President of Research.

Dr. Mitchell’s work in development and application of instruments and methods for determination of trace elements at unprecedentedly low levels in optical waveguide materials led to his induction into membership of the Materials Division of the National Academy of Engineering. He considers this one of the highlights of his career. Two of his other proudest achievements were his promotion to Director of the Materials Processing Research Laboratory at Lucent Technologies, Bell Laboratories, Murray, Hill NJ, and serving as Dean, College of Engineering and Architecture at Howard University in Washington, DC.

Throughout his career, Dr. Mitchell has contributed to production of future STEM scientists. He cofounded the Bell Laboratories Cooperative Research Fellowship Program that funded acquisition of Ph.D.s by 170 underrepresented minorities (URMs). He also founded the Bell Laboratories Summer Research Program for Minorities and Women that allowed nearly 1 800 junior college students to experience the lifestyle of a research scientist during a 10-week period. Each student was hosted by a Ph.D. research scientist/engineer.

Dr. Mitchell advises young people who want to succeed in STEM to follow the path he forged himself. He challenges them to

“Identify up to 10 heroes/sheroes and read their biographies. Because of their successes, envision yours.”

“Set specific goals and pursue them with passion. Along the way, define and obtain success at the personal level. Then exploit it to succeed in all aspects of your life.”

## What Sparked Their Individual Success?

### Primary and Secondary Education of the Authors

The authors grew up in the southern states of Louisiana, Alabama, and North Carolina. During their childhoods in the 1950s and early 1960s, they were legally educated in segregated schools.^1,2^ In retrospect, this was an advantage since their primary and secondary school teachers, Black and usually outstanding members of their communities, had a vested interest in their educational growth and worked closely with their parents to provide them with the best possible education. As a result, the authors, who were well-disciplined students, excelled scholastically with reinforcements from parents, teachers, and the community.

Each author attributes his success to his unique educational experiences, from elementary through graduate school. In contrast, the legally induced integrated K–12 schools that began in the late 1960s and early 1970s heaped considerable turmoil into the lives of many African American students who found themselves trying to learn in environments that were at best apathetic.^3,4^

During the authors’ formative years, forced segregation and later integration via busing was accompanied subsequently by considerable social and moral turmoil in this country. These situations, along with breakdown of the family structure and neighborhood school systems, contributed to a cataclysmic deterioration in the quality of public education for African Americans. As a result, many African Americans and other URM high school graduates are often deterred from receiving the foundation needed to achieve success in college in STEM curricula. To remedy this predicament, systemic transformation must occur in improvement of public education within metropolitan public schools. It is believed that mandatory, universal, free pre-K education will be one primary catalyst for advancement and improvement of urban K–12 school systems.

### Undergraduate Education

All three authors agree that attending an HBCU for their undergraduate education was key to their individual success due to the supportive environment and dedicated faculty they found at their respective institutions.^5,6^ While primarily White institutions (PWIs) were better funded and had superior facilities and resources, attending an HBCU provided the authors with extraordinary experiences that generated “success at the personal level” and the belief that they could accomplish any goal they pursued with commitment. Since most of their professors had earned their advanced degrees at PWIs and believed in their individual students’ abilities, these professors were able to prepare the authors for success in mainstream arenas. Thus, the authors believe that their respective HBCU professors had a deep commitment and additional incentives to encourage their future success. As an example, Professor Warner highlights the faculty at his undergraduate alma mater (Southern University) as having an ACS accredited department with 16 Black faculty with Ph.D.s from many of the top universities in this country.

### Graduate Education

The authors took similar but different routes to acquire their Ph.D.s. Following the completion of their B.S. degrees, Warner and May worked in the respective federal government contractor and federal government for several years, while Mitchell directly entered a Ph.D. program after his undergraduate education. Nevertheless, in each case, the authors sought to meet the same two major objectives for attending graduate school:

(1) acceptance by a university with a known reputation for successfully producing African American Ph.D.s in chemistry and
(2) obtaining funding via a teaching assistantship, fellowship, or research assistantship for their entire doctoral program.

Drs. Warner, May, and Mitchell, respectively, entered the University of Washington, University of Maryland, and Iowa State University and earned their respective Ph.D.s in 1977, 1977, and 1970. Selecting a chemistry department at a majority institution with a fundamental record of producing African American Ph.D.s was the best policy between 1965 and 1977, the period of time when these authors were in graduate school. In addition, the authors endorse this as the best guidelines for URM and female students to follow today. It is extremely important to research the track-record of the institution, the department, and even the professor, if possible, with respect to graduating a diverse group of Ph.D.s. PWIs committed to recruiting African American graduate students in STEM should have Black STEM alumni organizations and have developed booklets on the history of their Black graduates and faculty. Even without written records, such information is available through current and former Ph.D. students.

The three authors all attended PWIs for their graduate educations. All three selected advisors who were supportive and believed in their abilities. However, all three also had work experiences beyond their undergraduate degrees. Recall that Warner and May worked in contractor/government laboratories before graduate school, while Mitchell had a summer research internship at Oakridge National Laboratories before graduate school. All three believe that these work experiences helped to establish a level of confidence and independence in graduate school such that they were able to take charge of their graduate research early into their graduate careers and thus to perform well in graduate school.

## General Differences between HBCUs and PWIs

The authors assert that while desegregation greatly enhanced opportunities for Black students to have access to better facilities and greater funding for educational resources, it did not eliminate the racial attitudes of faculty or student bodies found in primarily white institutions (PWIs). Differences in quality of the environment of an African American undergraduate student at an HBCU and a desegregated state college/university can be depicted using the following actual example.

A Black student at an HBCU with an above average academic record would be sought automatically by several professors for academic and other opportunities (undergraduate research positions, summer jobs, etc.). At an integrated, majority school, an African American student with stellar credentials had to actively seek opportunities from a professor. Additionally, the student was subjected to an unreasonably intense scrutiny to justify his/her merit and prove her/his aptitude for an undergraduate research opportunity.

Even more important, the student at the HBCU would see/meet a significant number of African American professors whose mere existences provided living proof of the future that a student could envision for themselves. In addition, Black professors were willing to acknowledge to themselves and accept the fact that some of their students’ academic potential, intellect, and performance exceeded those of the professor at the same point in their careers. Consequently, because of a vested interest in their futures, the authors had committed mentors and role models. Extensive replications of these factors in PWIs have not yet been accomplished. However, if PWIs attain employee status approximating that of the U.S. population, their environment would begin to provide the same intangible and powerful advantages as HBCUs.

Fortunately, by the early 1990s, several majority universities had instituted exceptional undergraduate programs tailored to the success of all students, including African American students.^5−7^ These exemplary programs for African American undergraduates at PWIs often resulted from these institutions hiring full time diversity and minority administrators as well as from vigorous activism of Black students, Black faculty, and other committed students and faculty. For example, one of the first high-ranking diversity officer within a university setting occurred at the University of Washington in the late 1960s because of student activism.^8^

The authors also cite below an excellent example of changes in one of these well-known PWIs, i.e., the University of Maryland, Baltimore County (UMBC), whose African American President (Dr. Freeman Hrabowski) has championed changes on that campus through institution of campus programs, particularly his high performing Meyerhoff Scholars, which was initially for Black males only but later included Black females and then other under-represented groups.^9−11^ The excellent academic performance of these students at UMBC has enhanced the awareness and elevated high expectations of professors throughout PWIs within the U.S.

Despite these obvious advances at some PWIs, even today in 2022, HBCUs still provide distinct advantages for education of African Americans.^12^ Many of these institutions have enhanced their programs through efforts such as dual degree programs, regional university consortia, and through collaborative undergraduate summer programs with PWIs, industry, and government agencies.^13^

It is noteworthy that post segregation, HBCUs make up less than 5% of the U.S. college population. However, these institutions award more than 25% of the STEM bachelor’s degrees to Black students.^14^ In addition, according to the American Institute for Research, more than one-third of Blacks who received a STEM Ph.D. earned their undergraduate degrees at an HBCU.^15^

## Recommendations

It is our recommendation that known methods that have been demonstrated to work should be employed at all levels of education. For example, considerable efforts should be made toward recapturing the neighborhood school environments that were the pillars of success for Black students during the mid-1950s to late-1960s. This is not a recommendation for going back to segregation but rather a recommendation for trying to create an environment where teachers and schools care about all students and are willing to work with parents to promote the learning of all students. The potential role of pre-K in helping to accomplish this was cited earlier in this manuscript.

At the STEM undergraduate level, there should be a university consortium of collaborative undergraduate and summer programs that include universities, corporations, and federal and state government agencies. For example, NSF programs such as joint Engineering Research Centers (ERCs), Science and Technology Centers (STCs), and Centers of Research Excellence in Science and Technology (CREST) now provide HBCU students with access to better facilities and resources.^16^ The NSF stipulation for collaborations between these entities and HBCUs has had a profound effect.

For the 21st century and beyond, it is imperative that all educational systems, particularly the HBCU system, promote entrepreneurship by marrying B.S. degree education in STEM with business administration programs. This enhanced education/production of business owners, as well as traditional employees, is of critical and strategic importance for U.S. competitiveness and quality of life in this nation. According to the U.S. Department of Labor, only 5% of U.S. workers are employed in fields related to science and engineering, yet such workers are responsible for more than 50% of our sustained economic expansion.^18^

The worldwide impact of the HBCU system has been extraordinary since its inception in 1867, beginning with the founding of Howard University. The remarkable impact that HBCUs have achieved while being constrained by underfunding, less general resources, and limited facilities as compared to their majority counterparts should be recognized and enhanced by significant additional support. Current fiscal conditions at these institutions have produced a philosophy of “Do the best you can with what is available.” In contrast, majority PWIs have generally been able to plan the best possible programs/functions, while knowing they will be provided with funding for implementation. Within the 21st century, HBCUs should operate under the policy of “envision the best; conceive the best; create the best strategies; develop the best plans and obtain the funding needed to implement the best programs/functions”. This overall concept of operating institutions can best be sustained within a culture of excellence. Additionally, the authors believe that HBCUs should take the leading role of collaborating with underrepresented minority communities to develop STEM solutions for the major educational, economic, medical, and social problems confronting African American communities.

We note that gallant efforts have been periodically made to advance equality and inclusion of URMs and women in graduate chemistry programs in this country. NSF sponsored workshops constitute some of the most earnest efforts made in this arena.^19,20^ However, the authors are not aware of any easily available comprehensive and substantive follow-up studies to quantify the specific impact of these and other efforts. The authors strongly believe that standardized metrics should be developed to provide a guide for future success. For example, an accreditation-manual could be devised for U.S. academic STEM departments for self-evaluation and evolution of exemplary environments to promote and encourage excellence through diversity.

## Ph.D. Fellowships and Graduate Programs for URM and Women

Graduate programs that successfully produce African Americans with STEM Ph.D.s have increased from relatively few overall in the 1940s–1970s to islands of encouraging numbers during the 1980s and beyond. Professor Mitchell particularly notes the success of the AT&T-Lucent Technologies Bell Laboratories Cooperative Research Fellowship Program (CRFP) for URMs and the Graduate Research Program for Women (GRPW) for proving the equivalent excellence of the performance of URMs and women enrolled in the most highly rated universities in the United States.^21^ About 370 CRFP and GRPW graduates received Ph.D.s and became alumnus of such great institutions as MIT, Harvard, Berkeley, Princeton, Columbia, Yale, and others. These overwhelmingly successful Bell Laboratories programs also greatly dissipated the widely viewed myth that affirmative action programs to advance equity and increase the inclusion of African Americans and women mutually excluded the maintenance of excellence of employee performance. Many graduates of CRFP and GRPW were hired into the research areas of Lucent Bell Laboratories at Murray Hill, NJ; many others joined other distinguished agencies and corporations; 175 secured tenure track positions at U.S. universities; and many became the first African American or women to be tenured at their respective universities.^21^

To the best of the authors’ knowledge, the Bell Laboratories plan to produce a step function enhancement in the generation of outstanding URMs and women for participation in corporate and academic research has not been duplicated by any university sector to significantly increase the pool of candidates seeking careers as STEM professors. Should this become a national objective, appropriate task groups could be assembled to develop the strategy, define the resources, and implement the plan for achieving this goal.

Professor May notes the success of the program at NIST during his tenure there.^22^ He developed two programs focused on addressing the lack of underrepresented minority Ph.D.s.

**The Chemical Science and Technology Laboratory (CSTL) Graduate Fellowship Program** existed for 5 years and produced five Ph.D.s. Program participants were hired as NIST employees at the post baccalaureate level and required to have a 1-year period of residency before selecting and gaining admission to a graduate school of their choice. NIST paid their tuition and continued to pay their salary during this time. Upon graduation, candidates were expected to return to NIST and spend at least 1 year for each year they were in graduate school. One of the five has remained at NIST for more than 20 years.

The CSTL Graduate Fellowship Program gave way to the **Dolphus E. Milligan Graduate Fellowship Program** in 2006. The program, named after one of Dr. May’s first mentors at NIST, is a collaborative effort with the University of Maryland College Park, where applicants accepted into the graduate program are selected by NIST using a panel consisting of membership from the university and NIST with input from the Milligan Family. The program has resulted in the production of 17 Ph.D.s during its existence with another 5 in progress. Three are currently NIST professional staff members.

Professor Warner highlights the success of the graduate chemistry program at LSU, an institution that had previously been segregated well pass the middle of the 20th century. By the time professor Warner arrived in 1992, the Department of Chemistry had never had more than three African Americans working toward Ph.D.s at one time and had graduated its first African American Ph.D. in 1972. In fact, up until 1992, the department had graduated a total of six African American Ph.D.s from an institution that was more than a century old. Now, a recent independent publication^23^ has cited LSU Chemistry as leading the nation in number of African American Ph.D.s graduated, with a number approaching 100 as of the date of this publication. While the number for women was not quite as dismal as those for Blacks early on, that same publication cites LSU as leading the nation in the percentage of women who receive Ph.D.s in chemistry.

## Conclusions

The role of HBCUs in the education of Black Americans who pursue careers in STEM fields is still unique and irreplaceable. To propel further advancements for the future, significant additional funding should be provided to allow HBCUs to develop the best programs, to implement the best plans, and to sustain the best institutional functions. This implies that affordability of operating and sustaining an academic culture of excellence (ACE) should replace the historical one that entails “Do the best you can with the under-funding that is available.” is an imperative for the 21st century.

Advances in education and inclusion of underrepresented minorities and women Ph.D. research scientists and engineers at Lucent Technologies Bell Laboratories, Murray Hill, NJ. demonstrably refuted the previously widespread myth that a research organization cannot significantly improve the diversity of its staff and still maintain the high standards of qualifications for hiring and performance excellence of its employees. More than 300 URMs and women received their Ph.D. degrees from the most highly rated PWIs in the U.S. and pursued careers in Bell Laboratories, universities, corporations, and agencies.^21^

While the impact of a single individual on the diverse representation of an organization (department, division, or research area) can be significant, that influence ends with the absence of that singly committed individual. Instead for long-term progress, an entire organization must be reprogrammed and restructured from the ground up to the top level of the administration. This permits the “institutionalization of diverse excellence.” Even then, a decade of progress in a corporation can be eliminated by downsizing in a single year of recession. Fortunately, however, diverse excellence achieved in the workforce of an academic institution or government agency when institutionalized can also be sustainable.

Exemplary leadership roles exist for majority white institutions, HBCUs, Hispanic Serving Institutions, and minority STEM organizations to pursue solutions for the Grand Challenges confronting under-represented minorities. As a few examples, the memberships of the National Organization of Black Chemists and Chemical Engineers (NOBCChE), the Society for Advancement of Chicanos/Hispanics and Native Americans (SACNAS), and the American Chemical Society (ACS), both chemists and engineers are trained to scrutinize and solve such complex problems. To launch an appropriate attack on the Grand Challenges regarding STEM that confront America, we believe that analytical scientists may be relied upon to fill an important role. Finally, we ask “What role will you execute or what responsibility will you accept to contribute to the advancement and prosperity of future generations of STEM professionals?”

## Notes

This Perspective and Cover Art are part of *Analytical Chemistry*’s Diversity and Inclusion Cover Art Series.
